# Supplementary material for: Mapping nucleosome-resolution chromatin organization and enhancer-promoter loops in plants using Micro-C-XL
Source: Nat Commun. 2024 Jan 2;15:35. doi: 10.1038/s41467-023-44347-z (PMC10762229; doi:10.1038/s41467-023-44347-z)
Supplement: Supplementary file 1 — Supplementary Information [file 41467_2023_44347_MOESM1_ESM.pdf]

Supplementary Information File

Mapping nucleosome-resolution chromatin organization and enhancer-promoter loops in plants using Micro-C-XL

Linhua Sun, Jingru Zhou, Xiao Xu, Yi Liu, Ni Ma, Yutong Liu, Wenchao Nie, Ling Zou, Xing Wang Deng*, Hang He*

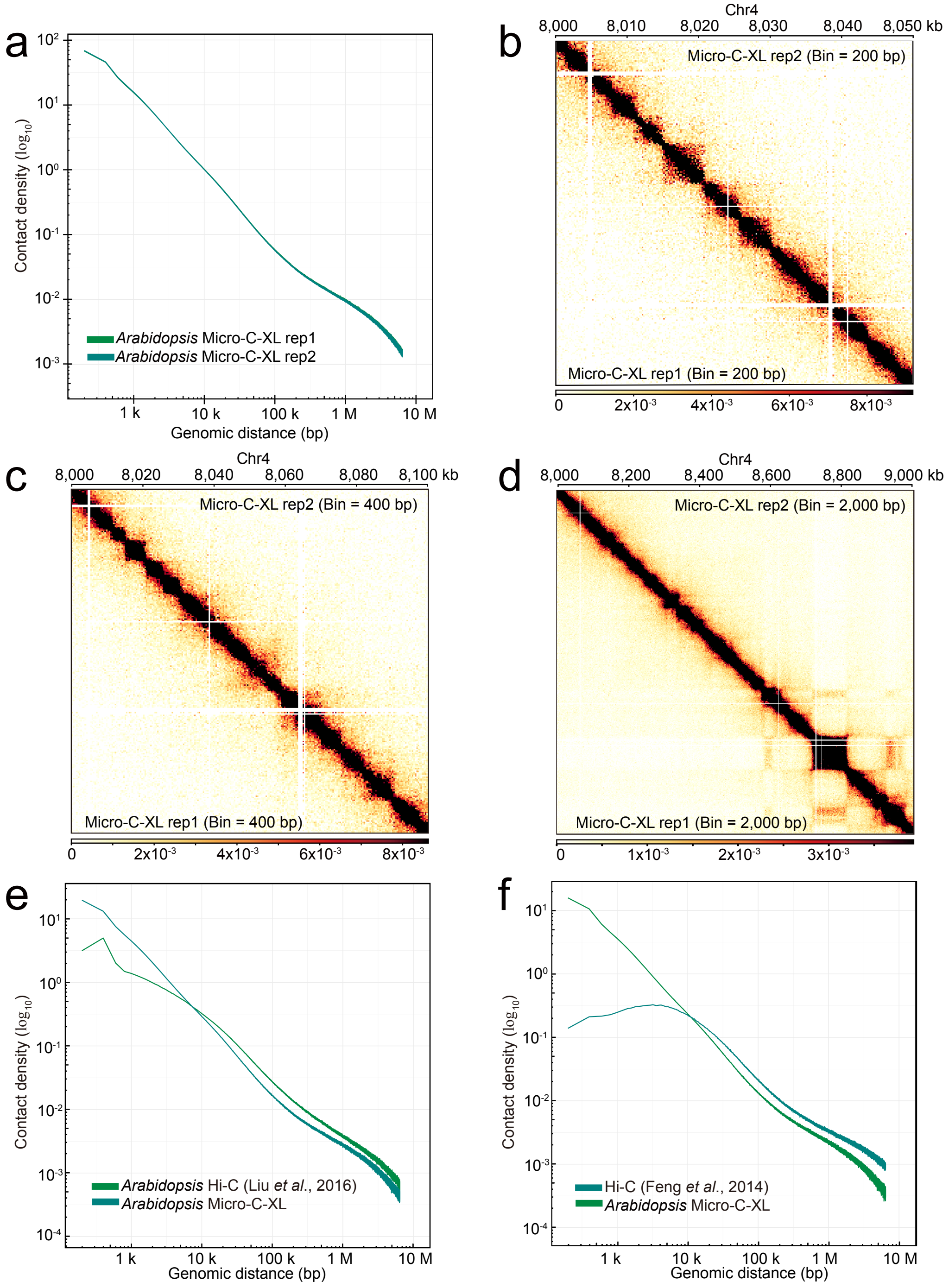

Supplementary Fig. 1 | *Arabidopsis* Micro-C-XL libraries have good reproducibility and quality.

a Scaling plot showing distance-dependent decay of contact density for *Arabidopsis* Micro-C-XL replicates 1 and 2, using 200-bp bins. **b** Asymmetrical contact map of typical loci showing good reproducibility in Micro-C-XL replicates 1 and 2 at 200-bp **b**, 400-bp **c,** and 2,000-bp **d,** resolutions. **e** Scaling plot showing distance-dependent decay of contact density for *Arabidopsis* Hi-C (Liu *et al.*, 2016, **e**) and Hi-C (Sun *et al.*, 2020, **f**) *v.s.* Micro-C-XL. Contact map of Micro-C-XL was downsampled to an equal number of valid counts, compared with Hi-C.

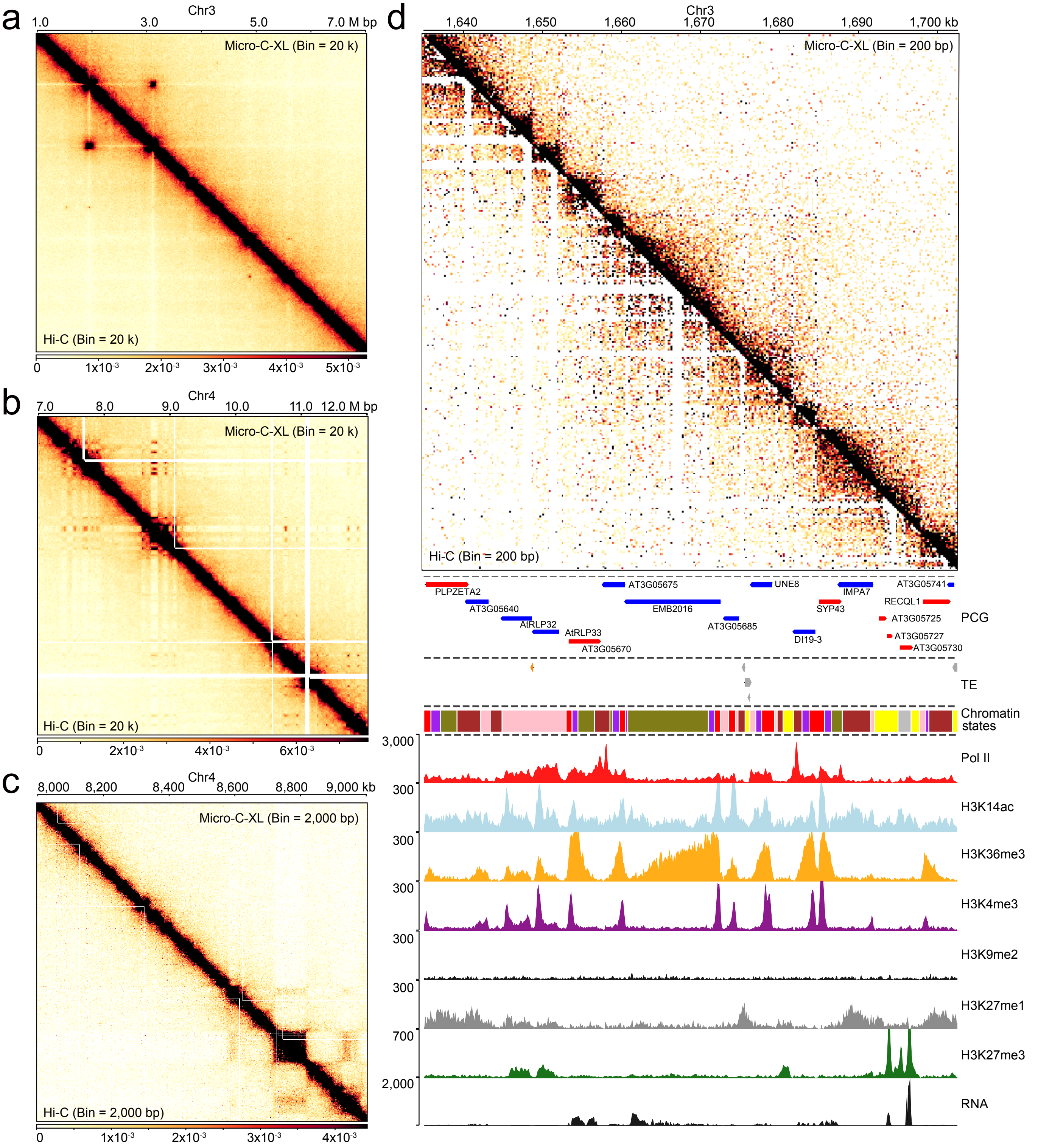

Supplementary Fig. 2 | Multiscale comparisons between Micro-C-XL and Hi-C.

a Asymmetrical contact map of typical loci for Hi-C (Sun *et al.*, 2020) and Micro-C-XL showing that Micro-C-XL detects typical chromatin organization in a manner similar to Hi-C **a**–**c**. **d** Asymmetrical contact map of typical loci for Hi-C (Sun *et al.*, 2020) and Micro-C-XL showing that Micro-C-XL outperforms Hi-C at nucleosome resolution (200-bp resolution) at the same number of valid counts. Contact map of Micro-C-XL was downsampled to an equal number of valid counts, compared with Hi-C.

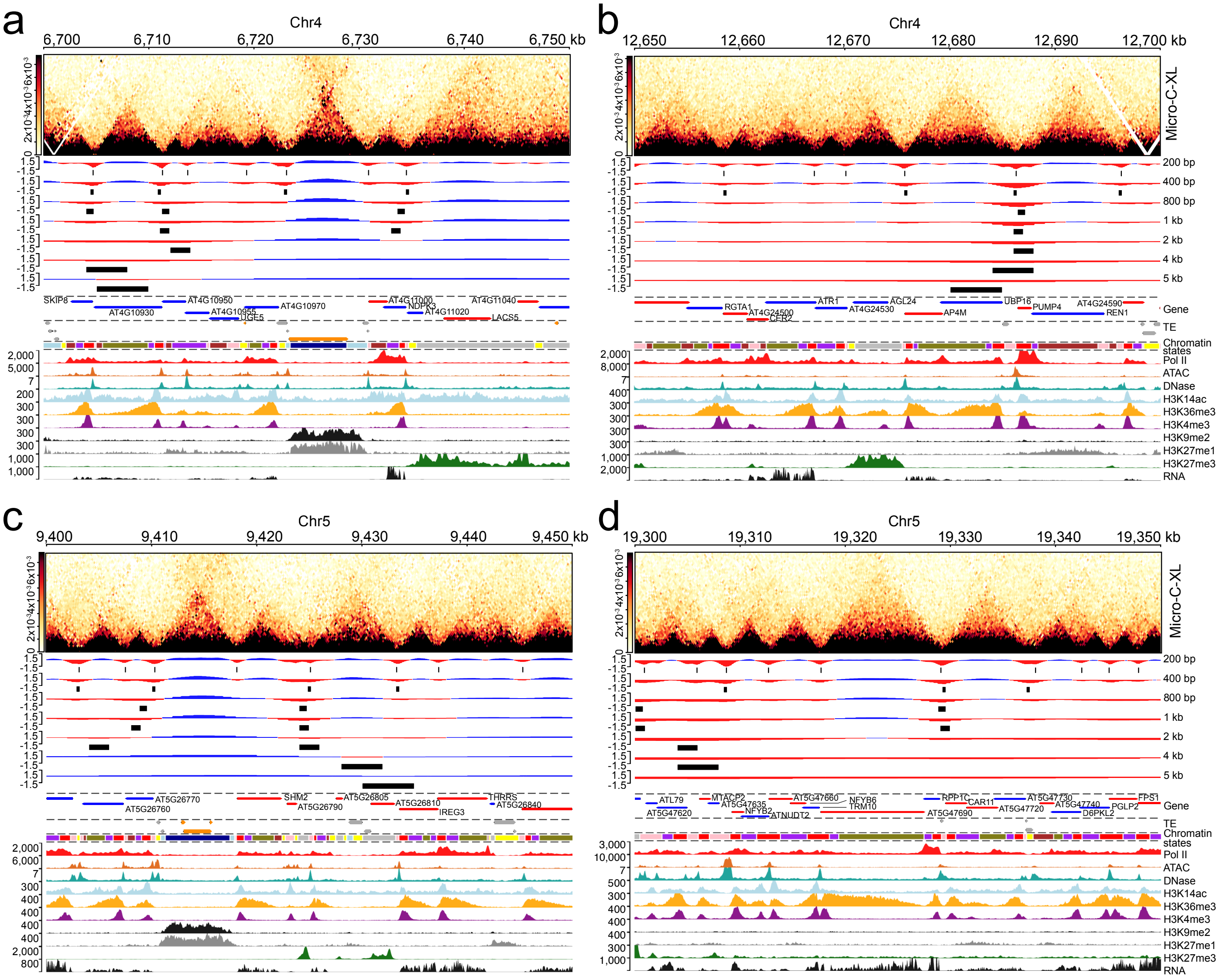

Supplementary Fig. 3 | Chromatin boundaries in the Micro-C-XL heatmap.

a Locus showing the insulation score, identified boundaries, and local chromatin organization patterns. Micro-C-XL heatmap showing chromatin interactions at 200-bp resolution. Insulation score distributions at multiple resolutions are shown after the heatmap. For each resolution, boundaries identified from the interaction matrix are indicated by black boxes. Tracks showing PCG annotations, TE annotations, chromatin states, Pol II occupancy, ATAC-Seq, DNase-Seq, multiple epigenetic modifications (e.g., H3K14ac, H3K36me3, H3K4me3, H3K9me2, H3K27me1, and H3K27me3), and RNA expression are presented under the insulation score tracks. The remaining three loci are shown in **b**–**d**.

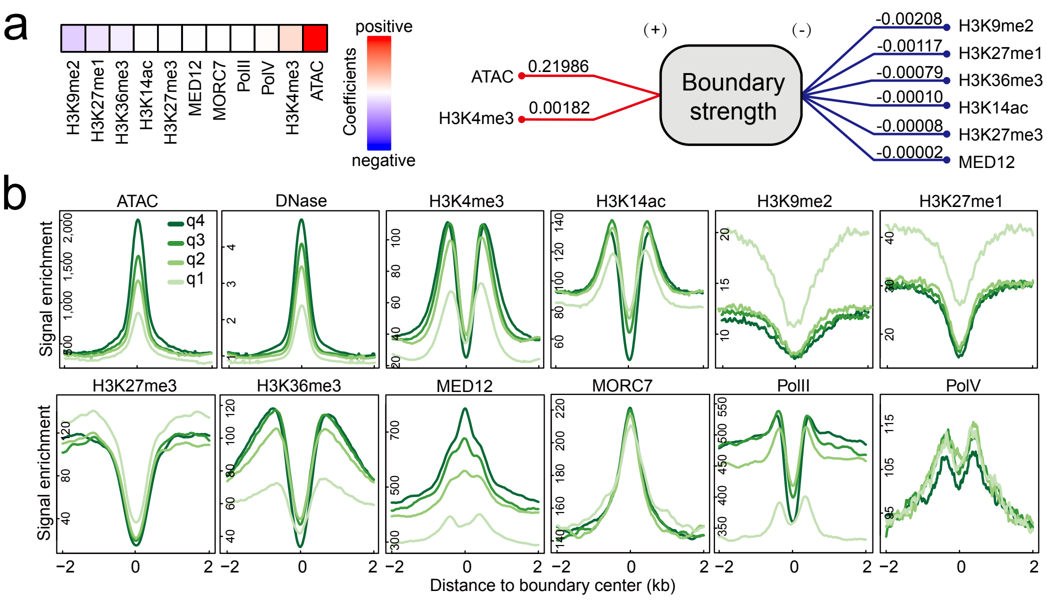

Supplementary Fig. 4 | Factors and chromatin boundaries.

a Heatmap of the regression coefficients from the generalized linear model for chromatin boundary strength prediction. The top parameters show positive and negative predictors of boundary strength through a schematic diagram (right). **b** Multiple ATAC-Seq/DNase-Seq/ChIP-Seq metagene plots over the boundary center. Chromatin boundaries were divided into four categories from weak to strong (q1 to q4) based on the boundary strength. The signal distribution of ATAC-Seq (or similarly DNase-Seq) increases proportionally with the distribution from q1 to q4, indicating that ATAC-Seq (or similarly DNase-Seq) is an excellent predictor of boundary strength.

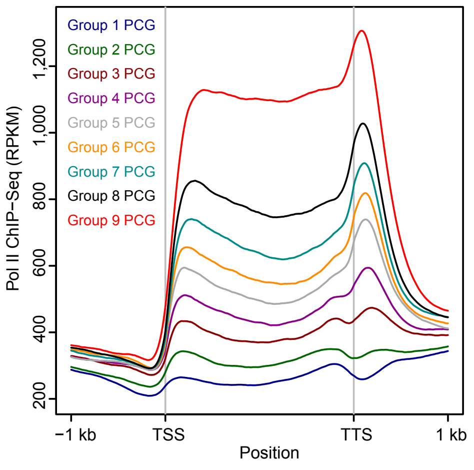

Supplementary Fig. 5 | Pol II enrichment across nine groups of PCGs.

Metagene plot showing enrichment of RNA Pol II occupancy across nine groups of PCGs (number ranges from 1 to 9, representing groups of genes with lowest to highest expression according to RNA-Seq).

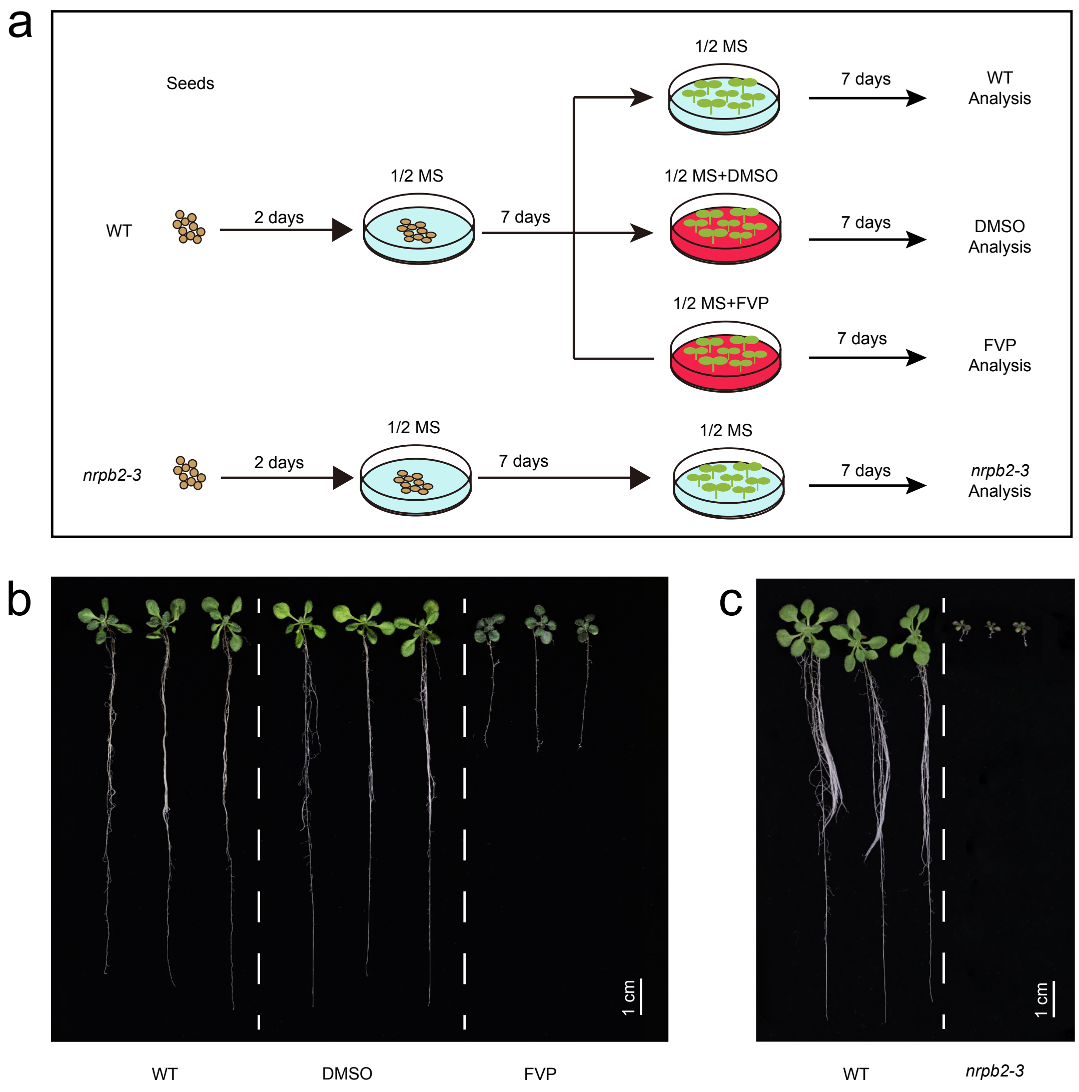

Supplementary Fig. 6 | *Arabidopsis* Pol II knockdown system.

a Flowchart showing *Arabidopsis* cultivation and processing. Our main objective was to treat *Arabidopsis* with the inhibitor (Flavopiridol, FVP) and detect the impact on chromatin structure. We adopted a postpone strategy by first culturing *Arabidopsis* wild type (WT) under normal conditions for 7 days and then transferring the seedlings to a medium containing FVP (also see Methods). Four groups of samples were collected, including WT, DMSO, FVP, and *nrpb2-3*, for subsequent experiments. **b** Morphological observation of *Arabidopsis* growth under different treatment conditions (WT, DMSO, and FVP). **c** Morphological observation of WT and *nrpb2-3*.

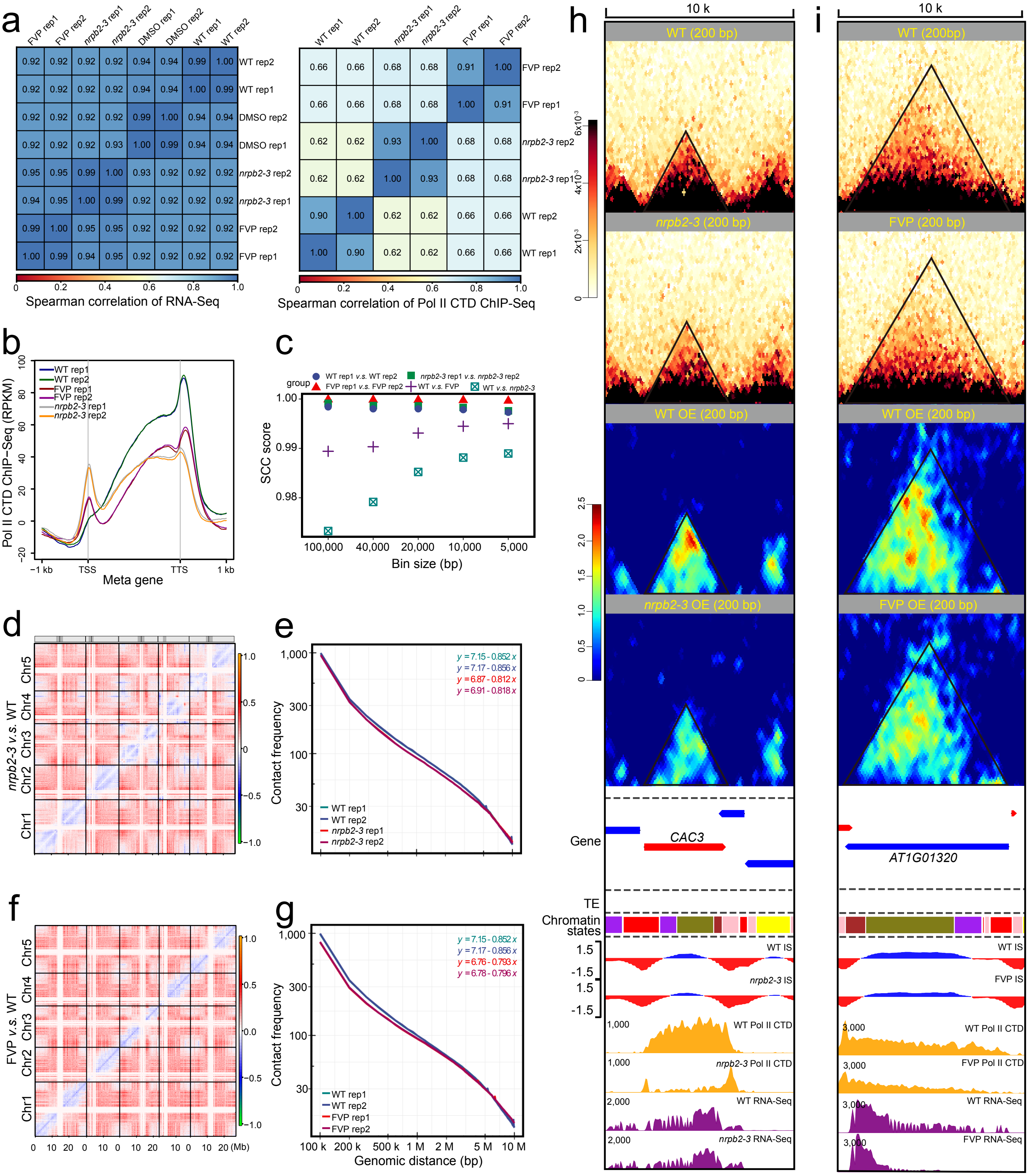

a
FVP rep1
FVP rep2
nrpb2-3 rep1
nrpb2-3 rep2
DMSO rep1
DMSO rep2
WT rep1
WT rep2
Spearman correlation of RNA-Seq
Spearman correlation of Pol II CTD ChIP-Seq
b
WT rep1
WT rep2
FVP rep1
FVP rep2
nrpb2-3 rep1
nrpb2-3 rep2
Pol II CTD ChIP-Seq (RPKM)
−1 kb
TSS
TTS
1 kb
Meta gene
c
group
WT rep1 v.s. WT rep2
nrpb2-3 rep1 v.s. nrpb2-3 rep2
FVP rep1 v.s. FVP rep2
WT v.s. FVP
WT v.s. nrpb2-3
SCC score
100,000
40,000
20,000
10,000
5,000
Bin size (bp)
d
nrpb2-3 v.s. WT
Chr5
Chr4
Chr3
Chr2
Chr1
e
y = 7.15 - 0.852 x
y = 7.17 - 0.856 x
y = 6.87 - 0.812 x
y = 6.91 - 0.818 x
Contact frequency
WT rep1
WT rep2
nrpb2-3 rep1
nrpb2-3 rep2
f
FVP v.s. WT
Chr5
Chr4
Chr3
Chr2
Chr1
(Mb)
g
y = 7.15 - 0.852 x
y = 7.17 - 0.856 x
y = 6.76 - 0.793 x
y = 6.78 - 0.796 x
Contact frequency
WT rep1
WT rep2
FVP rep1
FVP rep2
100 k
200 k
500 k
1 M
2 M
5 M
10 M
Genomic distance (bp)
h
10 k
WT (200 bp)
nrpb2-3 (200 bp)
WT OE (200 bp)
nrpb2-3 OE (200 bp)
Gene
CAC3
TE
Chromatin states
WT IS
nrpb2-3 IS
WT Pol II CTD
nrpb2-3 Pol II CTD
WT RNA-Seq
nrpb2-3 RNA-Seq
i
10 k
WT (200bp)
FVP (200 bp)
WT OE (200 bp)
FVP OE (200 bp)
AT1G01320
WT IS
FVP IS
WT Pol II CTD
FVP Pol II CTD
WT RNA-Seq
FVP RNA-Seq

Supplementary Fig. 7 | Alteration of Pol II can affect chromatin organization on both the large and small scales.

a Spearman correlation heatmaps for all RNA-Seq and ChIP-Seq samples. The repeatability between the biological replicates of each sample is good. **b** Metagene plots for Pol II CTD enrichment over all protein-coding genes in WT, *nrpb2-3*, and FVP. The signal value for each ChIP-Seq was normalized to their corresponding input. **c** Scatterplot summarizing the SCC values between biological replicates of each sample and between different samples, reflecting good repeatability. *nrpb2-3* and FVP treatment also impacted chromatin structure. **d** Relative difference interaction heatmaps showing the differences between WT and *nrpb2-3* (**d**) and between WT and FVP (**f**). **e** Decay plots showing the difference in chromatin interaction frequency between WT and *nrpb2-3* (**e**) and between WT and FVP (**g**). **h** Two other typical loci are shown: *nrpb2-3*-affected gene (**h**) and FVP-affected gene (**i**). Horizontal contact heatmap showing normalized chromatin interactions in WT and *nrpb2-3* (FVP in **i**) Micro-C-XL at 200-bp resolution. A black triangular box indicates a typical chromatin domain containing the entire single gene. Tracks showing PCG annotations, TE annotations, chromatin states, and insulation scores from Micro-C-XL, Pol II CTD ChIP-Seq, and RNA-Seq in WT and *nrpb2-3* (FVP in **i**) are displayed underneath. IS, insulation score.

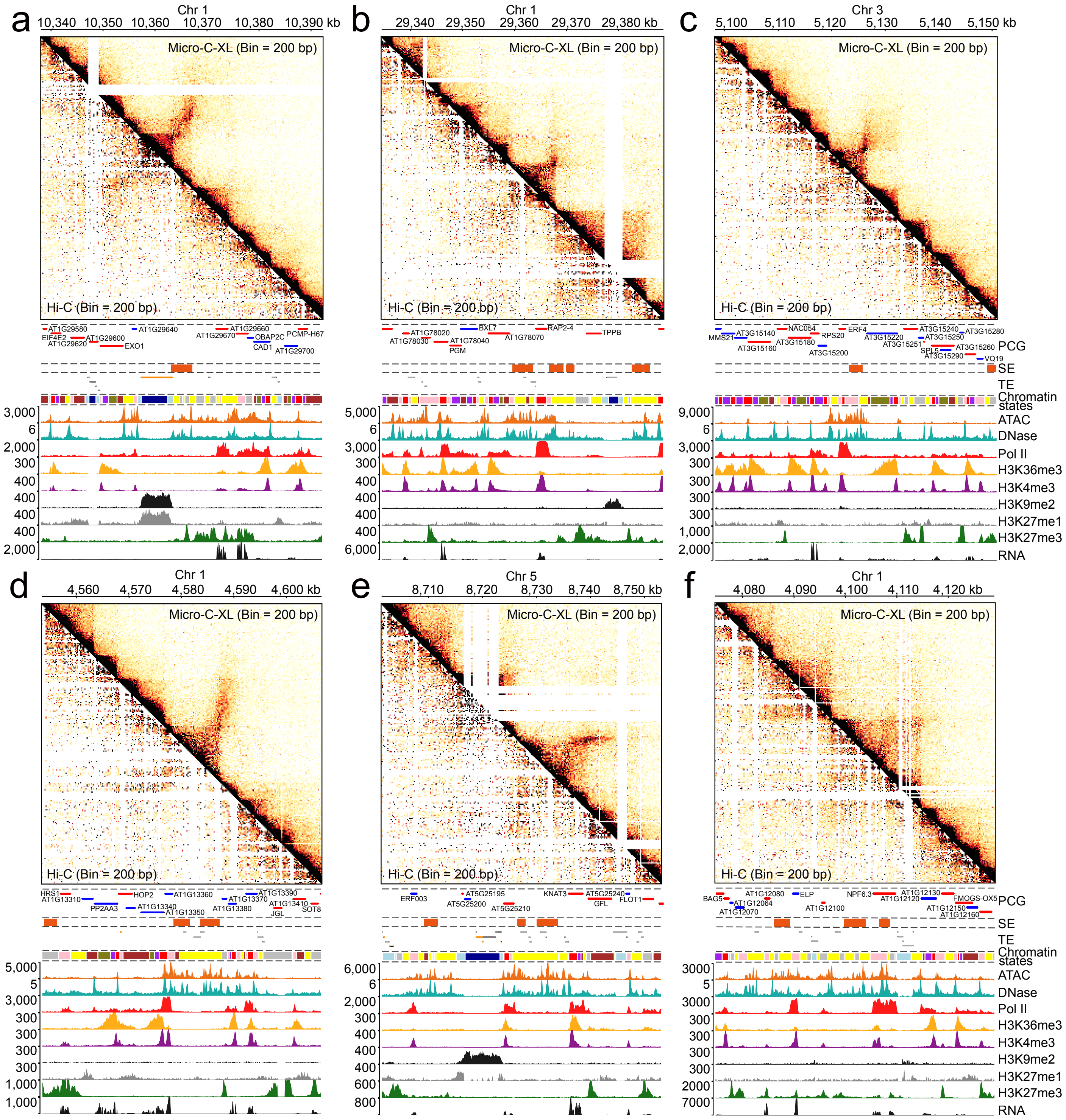

Supplementary Fig. 8 | Examples of extensive SE-associated chromatin loops, stripes, and domains.

a Asymmetrical heatmap showing chromatin interactions in Hi-C (left lower corner) and Micro-C-XL at 200-bp resolution (upper right corner). PCG, SE, TE, and chromatin states are presented under the heatmap. ATAC-Seq and DNase-Seq tracks are presented, along with tracks shown in previous figures. Heatmap of **(a–c)** showing typical loci containing chromatin loops between an SE and its target gene(s). **d, e** and **f** show other types of chromatin organization patterns: stripe and domain.

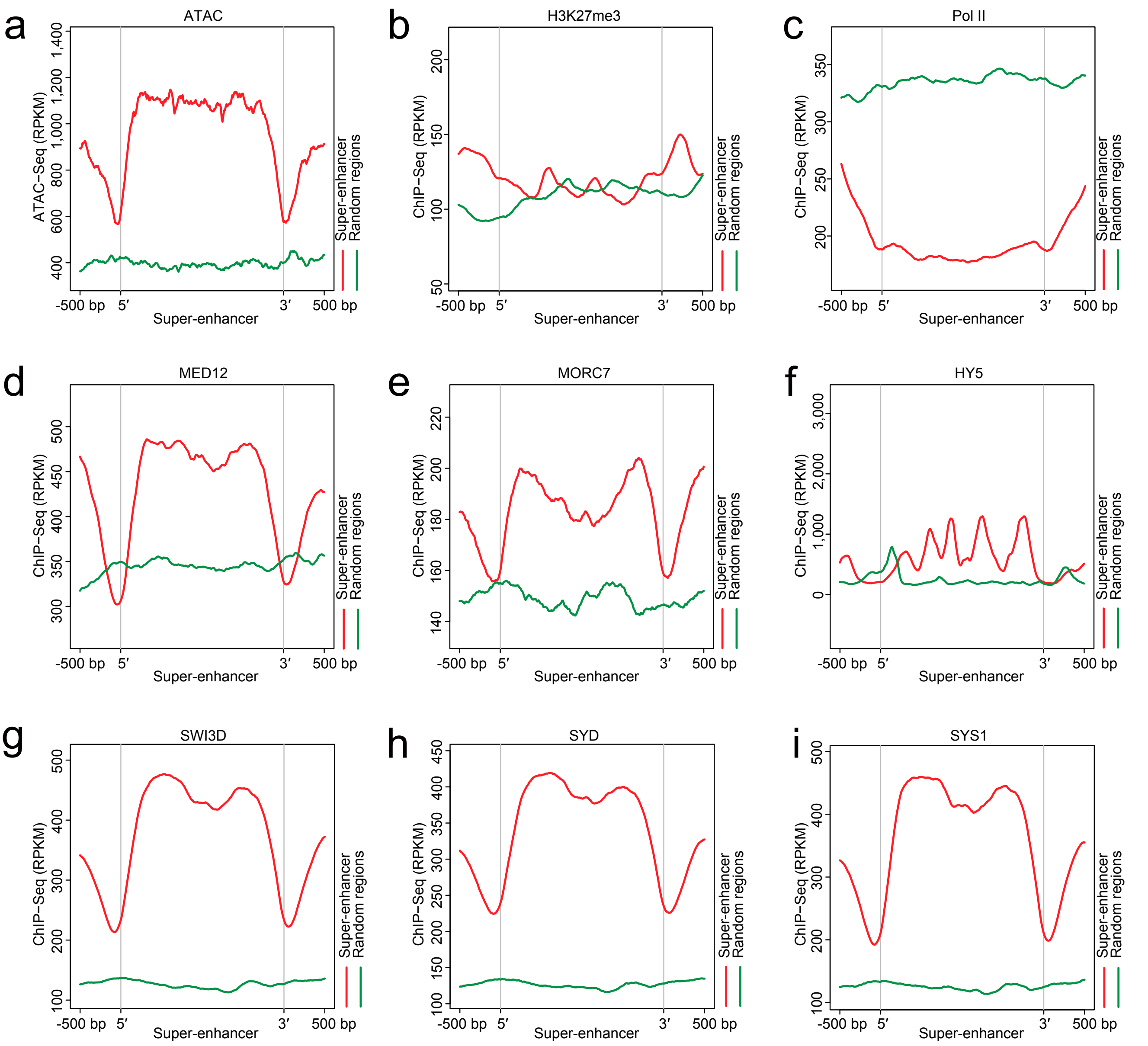

Supplementary Fig. 9 | Regulatory factors potentially involved in SE and SE-associated chromatin organization patterns.

a Metagene plot showing enrichment of ATAC-Seq reads coverage across SE regions as a positive control. **b** Metagene plot showing depletion of H3K27me3 reads coverage across SE regions as a negative control. **c-i** Other plots show enrichment of RNA Pol II (**c**), MED12 (**d**), MORC7 (**e**), SWI3D (**g**), SYD (**h**), and SYS1 (**i**) (but not HY5 (**f**)) across SE regions.

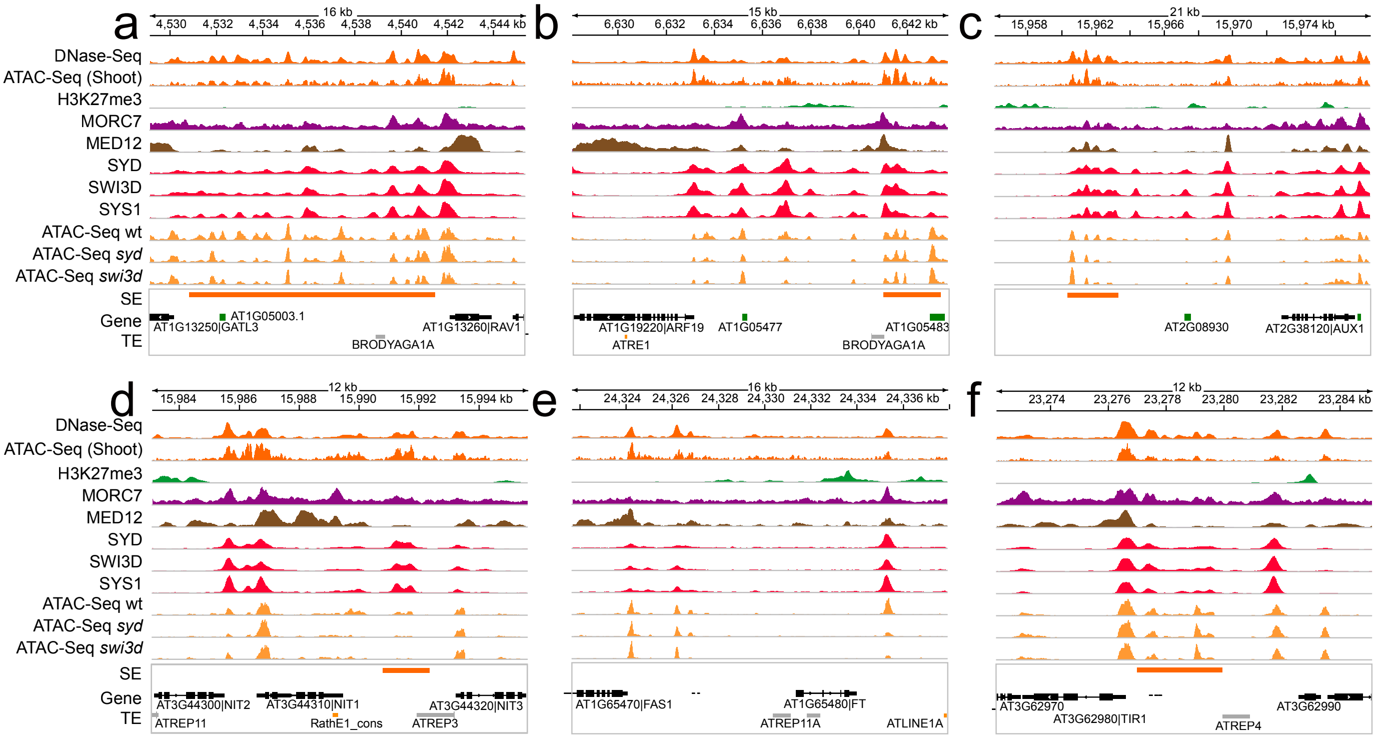

Supplementary Fig. 10 | Integrative Genomics Viewer screenshots showing typical loci of regulatory factors and SEs.

a Integrative Genomics Viewer screenshot showing distributions of DNase-Seq, ATAC-Seq, H3K27me3 ChIP-Seq, MORC7 ChIP-Seq, MED12 ChIP-Seq, SYD ChIP-Seq, SWI3D ChIP-Seq, SYS1 ChIP-Seq, and ATAC-Seq (WT, *syd*, and *swi3d*) at an SE. **b–f** Other typical loci. *FT* loci without SEs were used as control loci (**e**).

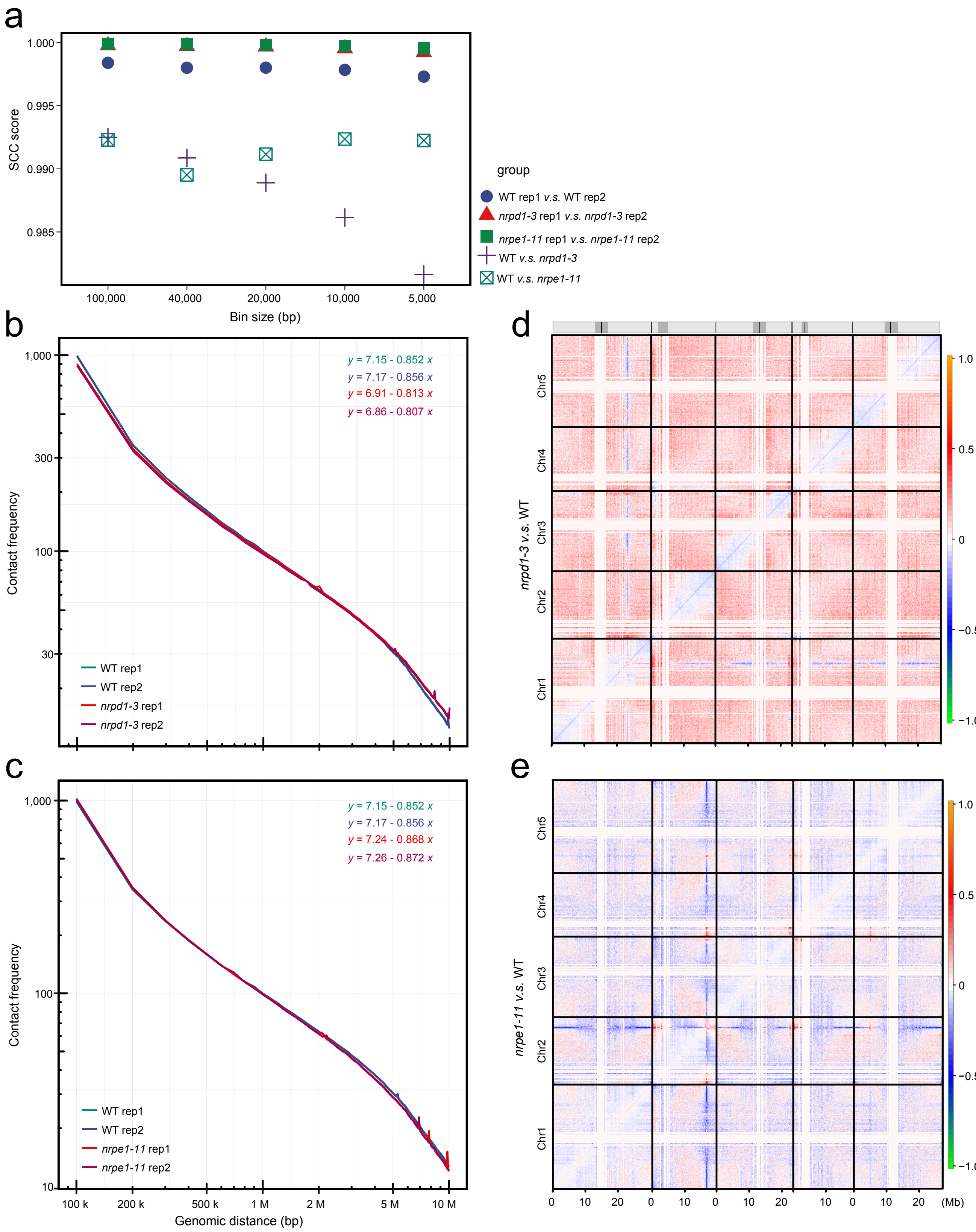
a
SCC score
1.000
0.995
0.990
0.985
100,000
40,000
20,000
10,000
5,000
Bin size (bp)
group
WT rep1 v.s. WT rep2
nrpd1-3 rep1 v.s. nrpd1-3 rep2
nrpe1-11 rep1 v.s. nrpe1-11 rep2
WT v.s. nrpd1-3
WT v.s. nrpe1-11
b
Contact frequency
1,000
300
100
30
y = 7.15 - 0.852 x
y = 7.17 - 0.856 x
y = 6.91 - 0.813 x
y = 6.86 - 0.807 x
WT rep1
WT rep2
nrpd1-3 rep1
nrpd1-3 rep2
c
Contact frequency
1,000
100
10
y = 7.15 - 0.852 x
y = 7.17 - 0.856 x
y = 7.24 - 0.868 x
y = 7.26 - 0.872 x
WT rep1
WT rep2
nrpe1-11 rep1
nrpe1-11 rep2
100 k
200 k
500 k
1 M
2 M
5 M
10 M
Genomic distance (bp)
d
nrpd1-3 v.s. WT
Chr5
Chr4
Chr3
Chr2
Chr1
1.0
0.5
0
−0.5
−1.0
e
nrpe1-11 v.s. WT
Chr5
Chr4
Chr3
Chr2
Chr1
1.0
0.5
0
−0.5
−1.0
0
10
20
0
10
0
10
20
10
0
10
20
(Mb)

Supplementary Fig. 11 | Quality control for Pol IV and Pol V mutants.

a Scatterplot summarizing the SCC values between biological replicates of each sample and between different samples, reflecting good repeatability. *nrpd1-3* and *nrpe1-11* treatments also impacted the chromatin structure. *nrpd1-3* and *nrpe1-11* are Pol IV and V key mutants, respectively. Data for WT rep1 *v.s*. WT rep2 is the same as in Supplementary Fig. 7c. **b** Decay plots showing the difference in chromatin interaction frequency between WT and *nrpd1-3* (**b**) and between WT and *nrpe1-11* (**c**). **d** Relative difference interaction heatmaps showing the differences between WT and *nrpd1-3* (**d**) and between WT and *nrpe1-11* (**e**).

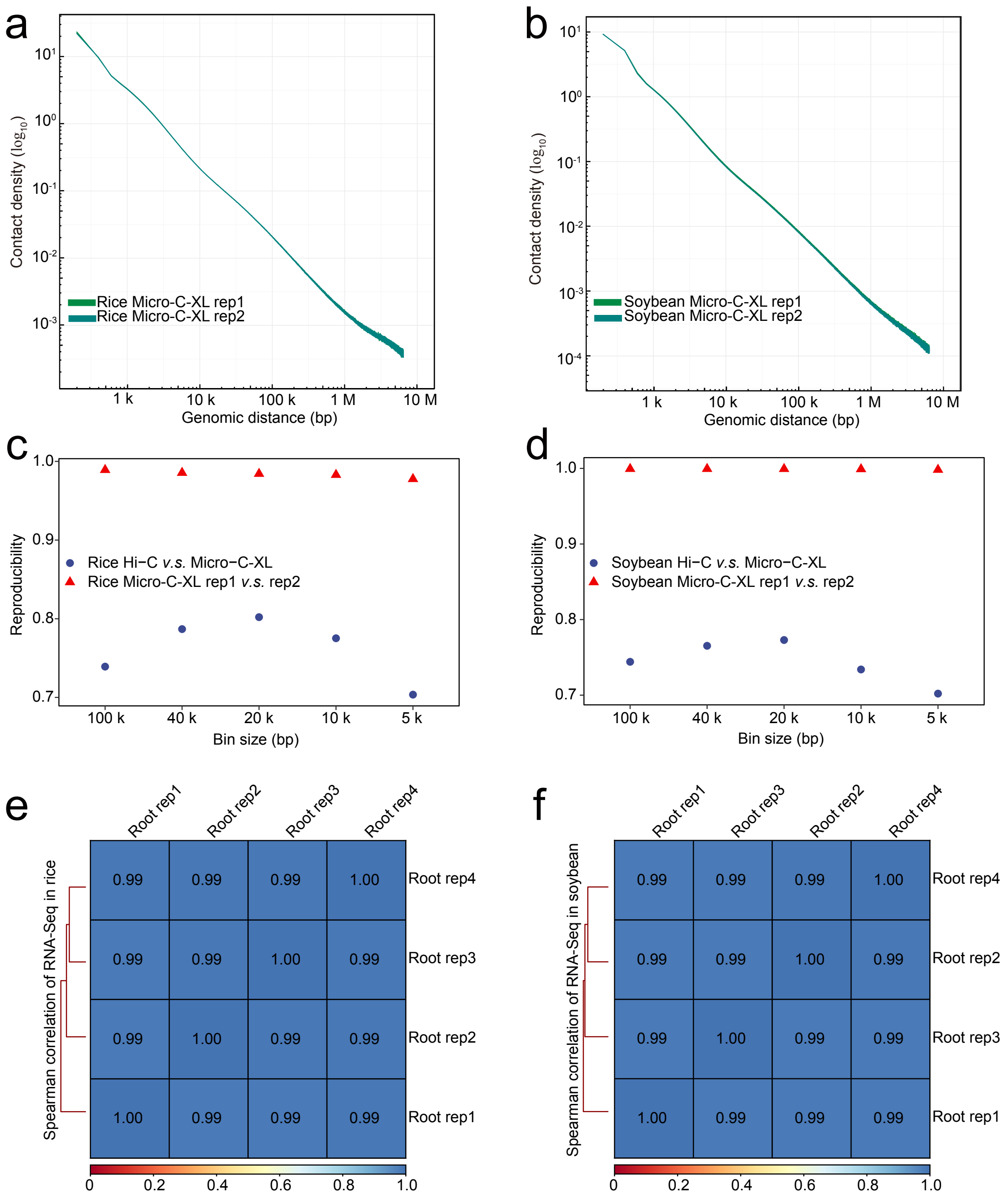

Supplementary Fig. 12 | Quality control of Micro-C-XL and comparison with Hi-C in rice and soybean.

a Scaling plot showing the distance-dependent decay of contact density for rice **(a)** and soybean **(b)** Micro-C-XL replicates 1 and 2, using 200-bp bins. **c** Comparison of reproducibility using HiCRep-measured SCC scores between rice Hi-C **(c)** (Gong *et al.*, 2018) and soybean Hi-C **(d)** (Wang *et al.*, 2021) *v.s.* newly generated Micro-C-XL (this study). Micro-C-XL maps were downsampled to equal valid counts, compared with Hi-C maps. **e** Spearman's correlation heatmap for RNA-Seq in rice and soybean **(f)**.
