# Supplementary material for: Mapping nucleosome-resolution chromatin organization and enhancer-promoter loops in plants using Micro-C-XL
Source: Nat Commun. 2024 Jan 2;15:35. doi: 10.1038/s41467-023-44347-z (PMC10762229; doi:10.1038/s41467-023-44347-z)
Supplement: Supplementary file 3 — Description of additional supplementary files [file 41467_2023_44347_MOESM3_ESM.docx]

Supplementary Data 1. Summary of Micro-C-XL quality control for all new samples.

Supplementary Data 2. *Arabidopsis* chromatin boundaries.

Supplementary Data 3. Public datasets used in this study.

Supplementary Data 4. Tables of *nrpb2-3-* and FVP-affected genes.
