# Supplementary material for: Mapping nucleosome-resolution chromatin organization and enhancer-promoter loops in plants using Micro-C-XL
Source: Nat Commun. 2024 Jan 2;15:35. doi: 10.1038/s41467-023-44347-z (PMC10762229; doi:10.1038/s41467-023-44347-z)
Supplement: Supplementary file 8 — Reporting Summary [file 41467_2023_44347_MOESM8_ESM.pdf]

nature portfolio

Corresponding author(s): Hang He

Last updated by author(s): Nov 28, 2023

Reporting Summary

Nature Portfolio wishes to improve the reproducibility of the work that we publish. This form provides structure for consistency and transparency in reporting. For further information on Nature Portfolio policies, see our Editorial Policies and the Editorial Policy Checklist.

Statistics

For all statistical analyses, confirm that the following items are present in the figure legend, table legend, main text, or Methods section.

n/a	Confirmed	
☐	☒	The exact sample size (*n*) for each experimental group/condition, given as a discrete number and unit of measurement
☐	☒	A statement on whether measurements were taken from distinct samples or whether the same sample was measured repeatedly
☐	☒	The statistical test(s) used AND whether they are one- or two-sided<br>*Only common tests should be described solely by name; describe more complex techniques in the Methods section.*
☒	☐	A description of all covariates tested
☒	☐	A description of any assumptions or corrections, such as tests of normality and adjustment for multiple comparisons
☒	☐	A full description of the statistical parameters including central tendency (e.g. means) or other basic estimates (e.g. regression coefficient) AND variation (e.g. standard deviation) or associated estimates of uncertainty (e.g. confidence intervals)
☐	☒	For null hypothesis testing, the test statistic (e.g. *F*, *t*, *r*) with confidence intervals, effect sizes, degrees of freedom and *P* value noted<br>*Give P values as exact values whenever suitable.*
☒	☐	For Bayesian analysis, information on the choice of priors and Markov chain Monte Carlo settings
☒	☐	For hierarchical and complex designs, identification of the appropriate level for tests and full reporting of outcomes
☐	☒	Estimates of effect sizes (e.g. Cohen's *d*, Pearson's *r*), indicating how they were calculated

Our web collection on statistics for biologists contains articles on many of the points above.

Software and code

Policy information about availability of computer code

Data collection	No software was used for data collection.
Data analysis	For Hi-C: fastp, HiC-Pro pipeline, coolers, and custom code from https://github.com/Linhua-Sun/Ath_Heat_Hi-C.<br>For Micro-C-XL: fastp, Dovetail Genomics pipeline (https://micro-c.readthedocs.io), CoolBox, coolpup.py, cooltools, ChIPseeker, and deeptools.<br>For ChIP-Seq and ATAC-Seq: Fastp, Bowtie2, Picard, SAMtools, deeptools, IGV, and SeqPlots.

For manuscripts utilizing custom algorithms or software that are central to the research but not yet described in published literature, software must be made available to editors and reviewers. We strongly encourage code deposition in a community repository (e.g. GitHub). See the Nature Portfolio guidelines for submitting code & software for further information.

Data

Policy information about availability of data

All manuscripts must include a data availability statement. This statement should provide the following information, where applicable:

- Accession codes, unique identifiers, or web links for publicly available datasets
- A description of any restrictions on data availability
- For clinical datasets or third party data, please ensure that the statement adheres to our policy

Raw Micro-C-XL/ChIP-Seq/RNA-Seq sequencing reads that support the findings of this study have been submitted to the National Genomics Data Center Genome Sequence Archive under accession number PRJCA014302. Hi-C, ChIP-Seq, ATAC-Seq, DNase-Seq, WGBS and RNA-Seq datasets used in this study are publicly available and summarized in Supplemental Data 3.

Research involving human participants, their data, or biological material

Policy information about studies with human participants or human data. See also policy information about sex, gender (identity/presentation), and sexual orientation and race, ethnicity and racism.

Reporting on sex and gender	n/a
Reporting on race, ethnicity, or other socially relevant groupings	n/a
Population characteristics	n/a
Recruitment	n/a
Ethics oversight	n/a

Note that full information on the approval of the study protocol must also be provided in the manuscript.

Field-specific reporting

Please select the one below that is the best fit for your research. If you are not sure, read the appropriate sections before making your selection.

[x] Life sciences [ ] Behavioural & social sciences [ ] Ecological, evolutionary & environmental sciences

For a reference copy of the document with all sections, see nature.com/documents/nr-reporting-summary-flat.pdf

Life sciences study design

All studies must disclose on these points even when the disclosure is negative.

Sample size	The sample size is determined according to the availability of data collection and in accordance with the data replication and statistical analysis standards and specifications.
Data exclusions	No data were exclued.
Replication	Micro-C-XL experiment of each species has two independent biological repeats.
Randomization	All samples were allocated randomly into experimental groups.
Blinding	The blinding design is not applicable to this system.

Reporting for specific materials, systems and methods

We require information from authors about some types of materials, experimental systems and methods used in many studies. Here, indicate whether each material, system or method listed is relevant to your study. If you are not sure if a list item applies to your research, read the appropriate section before selecting a response.

Materials & experimental systems

n/a	Involved in the study
☐	☒ Antibodies
☒	☐ Eukaryotic cell lines
☒	☐ Palaeontology and archaeology
☒	☐ Animals and other organisms
☒	☐ Clinical data
☒	☐ Dual use research of concern
☐	☒ Plants

Methods

n/a	Involved in the study
☐	☒ ChIP-seq
☒	☐ Flow cytometry
☒	☐ MRI-based neuroimaging

Antibodies

Antibodies used	Antibody in FVP, WT and nrpb2-3 samples(for ChIP-seq): RNA Pol II CTD antibody (mAb), Active Motif, 39097, 10 ul per ChIP added at a final dilution of 1:100<br>Antibody in input samples (for ChIP-seq): Goat anti-mouse IgG (H+L) antibody (HRP conjugate), EASYBIO, BE0102-100, 3 ul per ChIP added at a final dilution of 1:200
Validation	RNA pol II antibody (mAb), Active Motif, 39097: https://www.activemotif.com/catalog/details/39097/rna-pol-ii-antibody-mab.<br>Goat anti-Mouse IgG (H+L)-HRP conjugated, EASYBIO, BE0102-100:http://www.bioeasytech.com/product/2907.html?goods_id=5794

Dual use research of concern

Policy information about dual use research of concern

Hazards

Could the accidental, deliberate or reckless misuse of agents or technologies generated in the work, or the application of information presented in the manuscript, pose a threat to:

No	Yes
☒	☐ Public health
☒	☐ National security
☒	☐ Crops and/or livestock
☒	☐ Ecosystems
☒	☐ Any other significant area

Experiments of concern

Does the work involve any of these experiments of concern:

No	Yes
☒	☐ Demonstrate how to render a vaccine ineffective
☒	☐ Confer resistance to therapeutically useful antibiotics or antiviral agents
☒	☐ Enhance the virulence of a pathogen or render a nonpathogen virulent
☒	☐ Increase transmissibility of a pathogen
☒	☐ Alter the host range of a pathogen
☒	☐ Enable evasion of diagnostic/detection modalities
☒	☐ Enable the weaponization of a biological agent or toxin
☒	☐ Any other potentially harmful combination of experiments and agents

Plants

Seed stocks

All Arabidopsis, soybean, and rice plants used in this study were in Columbia-0 (Col-0), Willimas 82, and Oryza sativa L. (Nipponbare) backgrounds. nrpb2-3 was a kind gift from Dr. Binglian Zheng (Fudan University). nrpd1-3 (Salk_128428) and nrpe1-11 (Salk_029919) were provided by Dr. Weiqiang Qian (Peking University).

Novel plant genotypes

N/A

Authentication

N/A

ChIP-seq

Data deposition

☒ Confirm that both raw and final processed data have been deposited in a public database such as GEO.

☒ Confirm that you have deposited or provided access to graph files (e.g. BED files) for the called peaks.

Data access links
May remain private before publication.

Raw Micro-C-XL and other sequencing reads that support the findings of this study have been submitted to the National Genomics Data Center Genome Sequence Archive under accession number PRJCA014302. The data (bigwig files) reported in this paper have been deposited in the OMIX, China National Center for Bioinformation / Beijing Institute of Genomics, Chinese Academy of Sciences (https://ngdc.cncb.ac.cn/omix: accession no.OMIX004875).

Files in database submission

PolIICTDam_FVP_Input_R1.fq.gz
PolIICTDam_FVP_Input_R2.fq.gz
PolIICTDam_FVP_Rep1_R1.fq.gz
PolIICTDam_FVP_Rep1_R2.fq.gz
PolIICTDam_FVP_Rep2_R1.fq.gz
PolIICTDam_FVP_Rep2_R2.fq.gz
PolIICTDam_WT-Input_R1.fq.gz
PolIICTDam_WT-Input_R2.fq.gz
PolIICTDam_WT-Rep1_R1.fq.gz
PolIICTDam_WT-Rep1_R2.fq.gz
PolIICTDam_WT-Rep2_R1.fq.gz
PolIICTDam_WT-Rep2_R2.fq.gz
PolIICTDam_nrpb2-3_Input_R1.fq.gz
PolIICTDam_nrpb2-3_Input_R2.fq.gz
PolIICTDam_nrpb2-3_Rep1_R1.fq.gz
PolIICTDam_nrpb2-3_Rep1_R2.fq.gz
PolIICTDam_nrpb2-3_Rep2_R1.fq.gz
PolIICTDam_nrpb2-3_Rep2_R2.fq.gz
PolIICTDam_FVP_Input.bw
PolIICTDam_FVP_Rep1.bw
PolIICTDam_FVP_Rep2.bw
PolIICTDam_WT-Input.bw
PolIICTDam_WT-Rep1.bw
PolIICTDam_WT-Rep2.bw
PolIICTDam_nrpb2-3_Input.bw
PolIICTDam_nrpb2-3_Rep1.bw
PolIICTDam_nrpb2-3_Rep2.bw

Genome browser session
(e.g. UCSC)

The data (bigwig files) reported in this paper have been deposited in the OMIX, China National Center for Bioinformation / Beijing Institute of Genomics, Chinese Academy of Sciences (https://ngdc.cncb.ac.cn/omix: accession no.OMIX004875).

Methodology

Replicates

ChIP-Seq experiment of each species has two independent biological repeats.

Sequencing depth

	Total reads	Uniq mapped reads	Reads length	Ends
PolIICTDam_WT-Rep1	35,895,316	31,851,649	150	paired
PolIICTDam_WT-Rep2	30,533,902	27,157,360	150	paired
PolIICTDam_WT-Input	38,625,060	27,128,850	150	paired

	PolIICTDam_nrpb2-3_Rep1 44,055,196 40,822,559 150 paired<br>PolIICTDam_nrpb2-3_Rep2 43,565,440 40,671,111 150 paired<br>PolIICTDam_nrpb2-3_Input 48,075,872 30,944,906 150 paired<br>PolIICTDam_FVP_Rep1 50,292,128 47,214,085 150 paired<br>PolIICTDam_FVP_Rep2 46,324,006 43,685,592 150 paired<br>PolIICTDam_FVP_Input 46,031,244 33,520,398 150 paired
Antibodies	Antibody in FVP, WT and nrpb2-3 samples: RNA Pol II CTD antibody (mAb), Active Motif, 39097, 10 ul per ChIP added at a final dilution of 1:100<br>Antibody in input samples: Goat anti-mouse IgG (H+L) antibody (HRP conjugate), EASYBIO, BE0102-100, 3 ul per ChIP added at a final dilution of 1:200
Peak calling parameters	MACS2: -f BAM -g 119146348 -q 0.01
Data quality	All identified peaks in this study were called with q-value threshold as 0.01. PCA from deeptools was used for QC of multiple ChIP-Seq samples.
Software	bowtie2<br>Samtools<br>Picard's MarkDuplicates<br>deeptools<br>MACS2<br>bedtools<br>Integrative Genomics Viewer<br>SeqPlots
